# Root-Zone Temperature Drives Coordinated Photosynthesis, Root Architecture, and Metabolism Responses in *Schisandra chinensis* (Trucz.) Baill

**DOI:** 10.3390/plants14162595

**Published:** 2025-08-20

**Authors:** Huimin Tang, Xiaoqian Song, Lu Jin, Weisan Zhang, Jie Zheng, Lu Zhang, Qiuyu Yu, Yu Shi, Xin Guan, Zhonghua Zhang, Chunying Zheng, Zhonghua Tang

**Affiliations:** 1 College of Chemistry, Chemical Engineering and Resource Utilization, Northeast Forestry University, Harbin 150040, China; tanghuimin19@163.com (H.T.);; 2 Key Laboratory of Forest Plant Ecology, Northeast Forestry University, Ministry of Education, Harbin 150040, China; 3 School of Life Sciences, Heilongjiang University, Harbin 150040, China; zhengchunying68@163.com

**Keywords:** root-zone temperature, *Schisandra chinensis* (Turcz.) Baill., lignans, phenolic compounds, HPLC, LC-MS

## Abstract

Soil warming due to climate change has a significant potential impact on crop yield and quality. *Schisandra chinensis* (Trucz.) Baill, a multipurpose plant disseminated in the highly climate-sensitive region of Northeast Asia, is affected by soil warming, which limits the supply and quality of raw materials. This study investigated the differential responses of biomass accumulation and bioactive ingredient production across various organs to root-zone temperature (RZT) variations, employing both physiological assessments and metabolomic profiling. Elevated root temperatures may increase plant biomass and indirectly increase photosynthetic rates by promoting root growth; however, biomass responses differ among organs. A 20 °C root temperature promoted stem and leaf growth and inhibited root development, whereas a 30 °C root temperature significantly promoted root growth but reduced leaf biomass. Schisanhenol A, a key bioactive lignan serving as a quality marker for *S. chinensis*, displayed synthesis dependent on temperature. Concurrently, flavonoid biosynthesis is coordinated accumulation at the naringenin nodal point. A 15 °C RZT inhibited lignan production in roots while triggering stress-responsive phenol accumulation in leaves (41.39%). Conversely, at 20 °C and 30 °C RZTs, schisanhenol synthesis was repressed in leaves but accumulated in roots (9.8–25.71%). It is worth noting that the increase in RZT significantly promoted the synthesis and accumulation of schisandrol A in the aboveground part of the plant (43.88%). This research underscores that a suitable elevation in root-zone temperature can augment the medicinal attributes of the aerial components of *S. chinensis*.

## 1. Introduction

*Schisandra chinensis* (Turcz.) Baill. *(S. chinensis)* is a phytotherapeutic agent of significance in traditional Chinese medicine, as well as in the traditional pharmacopeias of Korea, Russia, and Japan. Phytochemical analyses confirm this species to be a rich source of bioactive compounds, including lignans, triterpenoids, phenolic acids, flavonoids, organic acids, vitamins, and polysaccharides [1]. Among the bioactive constituents, lignans are the characteristic bioactive compounds. Pharmacological investigations have demonstrated its broad-spectrum therapeutic efficacy, including having cardioprotective, hepatoprotective, antioxidant, hypoglycemic, antimicrobial, anti-inflammatory, and antitumor effects, as well as enhancing the ability to sleep, remember, and concentrate [2,3,4]. Phytochemical profiling also revealed lignan presence in both aglycone and glycoside configurations across all organs, including the stems, leaves, and roots of the plant [1]. Increasing demand for effective disease treatments, coupled with growing interest in cosmetics, functional foods, and diversified food sources, has driven greater utilization of the non-fruit plant parts (stems, leaves, and roots) of *S. chinensis* [5]. Current industrial utilization has expanded to include functional food engineering, particularly in the development of specialty products such as leaf teas, bioactive beverages, nutraceuticals, and phytochemical-enriched condiments [6,7]. This growing demand is further propelled by an expanding range of applications and a heightened focus on the sustainable sourcing of raw materials.

Lignans have been confirmed to be the most bioactive constituents of *S. chinensis*. Comprehensive investigation has led to the isolation of nearly 200 lignans from *S. chinensis* plants. Thorough analysis of the current literature identifies schisandrol A, schisandrol B, schisantherin B, schizandrin A, schizandrin B, schizandrin C, and schisantherin A as particularly significant among these lignans. Notably, schisandrol A has emerged as a pivotal marker compound for quality evaluation [8,9]. Lignans are widely distributed across plant tissues, exhibiting concentrations in vegetative organs (roots, stems, leaves) comparable to or exceeding those found in the fruits [10,11]. Given the limitations of fruit-derived lignans in meeting market demand, *S. chinensis* roots, stems, and leaves are emerging as promising alternative sources. The tissues under consideration are abundant in bioactive compounds, particularly lignans. This characteristic renders them ideal candidates for the development of functional foods and dietary supplements. Lignans are synthesized in a separate branch of the phenylpropanoid pathway [12,13,14]. The biosynthesis pathway of phenylpropane is initiated by phenylalanine metabolism, yielding lignin, flavonoids, and phenolic compounds as its byproducts. It has been determined that this pathway is of significant importance in the synthesis of secondary metabolites and the strength of plant resistance. The chemical composition and content of the compounds are subject to variation due to their growth characteristics and environmental factors, including temperature, light intensity, and precipitation. Therefore, further research into the environmental factors influencing the bioactive compound content is essential. This research is essential for enhancing the quality of locally produced *S. chinensis* for use as a functional raw material.

Climate change has increased the frequency of extreme high-temperature events, such as heat waves, adversely impacting plant development and agricultural productivity through exacerbated thermal stress [15,16]. *S. chinensis* inhabits northeastern China, the Russian Far East, and the Korean Peninsula, regions characterized by seasonally frozen soils and cold-adapted ecosystems. In contrast to variable aboveground air temperatures, the root-zone soil temperature displays significant thermal inertia, allowing aberrant states to persist more readily [17]. Roots are highly sensitive to their soil environment, and temperature significantly influences plant growth and development [18]. Root-zone temperature (RZT) is a pivotal soil factor governing root growth and function, significantly influencing plant growth, root physiology, and assimilate distribution [19,20]. Suboptimal RZTs impede shoot and root biomass accumulation, even when aerial tissues experience optimal temperatures [21]. Elevated temperatures can adversely affect key physiological processes, including photosynthesis, respiration, water balance, and membrane stability [22]. In response to root-zone temperature changes, plants enhance physiological performance by modulating secondary metabolism to maintain yield [23,24]. The production of plant secondary metabolites is closely related to the secretion of RZT. However, in contrast to extensive research on the temperature responses of shoots, the soil temperature response of roots is still relatively unexplored [20,24,25]. We conducted experiments partitioning the temperature between the aerial and root tissues to evaluate the effects of soil warming on *S. chinensis* growth, development, and the synthesis of lignans and flavonoids across different organs, including their metabolic coordination.

## 2. Results and Analysis

### 2.1. Effects on Plant Growth of Different RZT Treatments

**Figure 1:**
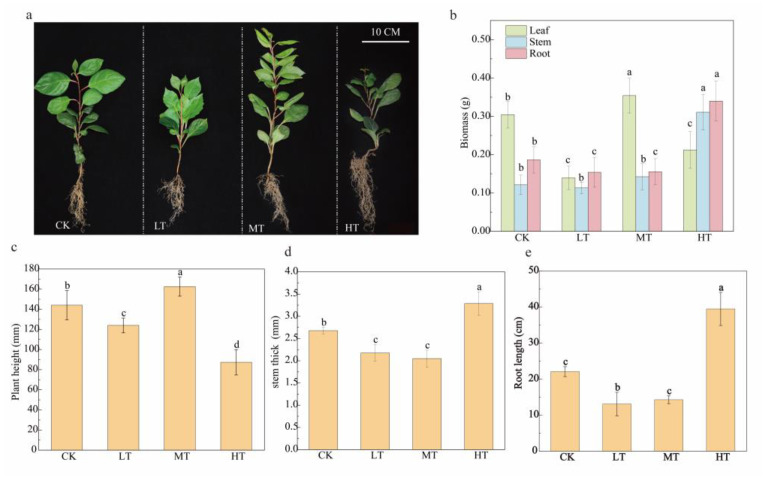
Responses of morphology (**a**), biomass (**b**), plant height (**c**), stem thickness (**d**), and root length (**e**) of *S. chinensis* to temperatures in the root zone. Values are mean and standard error of six repeated measurements. Small letters indicate significant differences (*p* < 0.05).

Following 30-day exposure to varying root-zone temperatures, morphological and growth differences in *S. chinensis* plants were observed (Figure 1a). Compared to the controls (25 °C), plants exposed to a 15 °C root-zone temperature showed significant reductions in plant height, root length, and stem thickness. At a 20 °C RZT, *S. chinensis* showed the greatest increase in plant height, although both the basal diameter and the root length remained significantly lower than the control values. Plants subjected a 30 °C RZT (HT) exhibited significantly greater stem thickness and root length than plants in other temperature groups, whereas plant height was significantly lower than that in the 15 °C (LT) treatment. Root biomass under HT conditions was significantly greater than in other treatments (*p* < 0.05).

**Figure 2:**
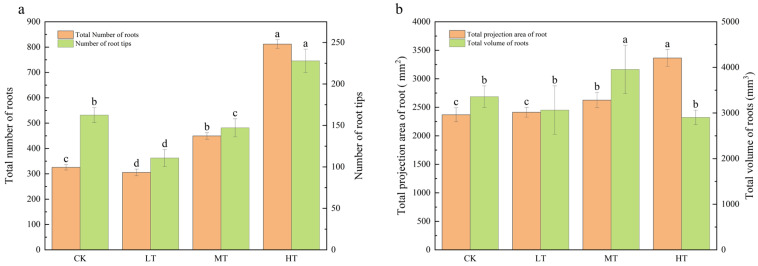
*S. chinensis* (**a**) total root number and root tip number and (**b**) root projection area and total root volume response to different root zone temperatures. Values represent the mean and standard error of six replicates. Different smaller letters indicate that there are significant differences in *S. chinensis* among the different root-zone temperature treatment groups (*p* < 0.05).

Relative to the control (25 °C), the number of root tips in *S. chinensis* exhibited temperature-dependent variation: a 32.31% reduction at 15 °C, a 9.45% reduction at 20 °C, and a 39.69% increase at 30 °C (Figure 2a). The root-zone temperature significantly influenced the total root count, showing 38.02% and 149.28% enhancements at 20 °C and 30 °C, respectively, relative to the controls. The 30 °C treatment induced a 42.01% greater root projection area compared to controls, while the 20 °C treatment increased root volume by 17.81% (Figure 2b). It was found that the low-temperature treatment (15 °C) inhibited the root development process, characterized by a significant reduction in total root number, projected area, volume, and root-tip density.

### 2.2. Response of Gas Exchange Under Different RZTs

The impact of root-zone temperature on the photosynthetic characteristics of *S. chinensis* seedlings was investigated. On day 30, Pn, Tr, and Gs values were significantly elevated relative to the control; however, Gs showed no significant difference between treatments and remained stable. In contrast, Ci exhibited distinct variation patterns compared to other photosynthetic parameters (Table 1). We also investigated the influence of the root-zone temperature on the levels of photosynthetic pigments (e.g., chlorophyll and carotenoids). Root-zone temperature treatments increased the content of photosynthetic pigments compared to the control. Chlorophyll a, chlorophyll b, and carotenoid contents were consistently higher than those in the control. At the same time, the photosynthetic pigment content gradually increased with increasing root-zone temperature. At 15 °C and 20 °C RZTs, the photosynthetic pigment content showed slight, non-significant elevations.

### 2.3. Response of Chlorophyll Fluorescence Level Under Different RZT Conditions

**Figure 3:**
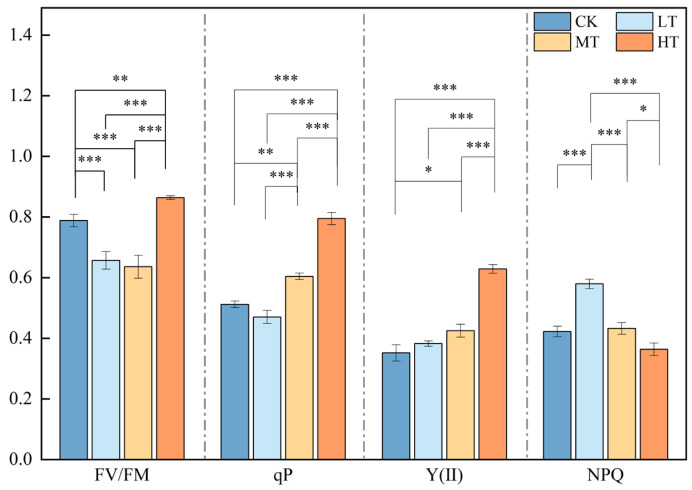
Chlorophyll fluorescence level changes in *S*. *chinensis* under different RZT conditions. Statistical analyses were performed using one-way ANOVA, *p* < 0.05, followed by the LSD test. Values are expressed as means ± SD (*n* = 5). Stars (* *p* < 0.05, ** *p* < 0.01, *** *p* < 0.001) above the connecting lines indicate significance levels between different RZT treatments.

The chlorophyll fluorescence characteristics of *S. chinensis* varied significantly under different root-zone temperature conditions, with some treatments exhibiting significant changes. Low root-zone temperatures resulted in photoinhibition of PSII in *S. chinensis* leaves. Specifically, the Fv/Fm of PSII in *S. chinensis* leaves decreased by 16.66% and 19.33% in the 15 °C and 20 °C low-temperature treatments, respectively, both values reaching statistical significance (*p* < 0.05; Figure 3). The photochemical quenching coefficient (qP) increased by 18% and 55.34% compared to CK at RZTs of 20 °C and 30 °C, respectively, while no significant change occurred at an RZT of 15 °C. Furthermore, compared to CK, the PSII quantum yield [Y(II)] of leaves increased by 28.85% and 78.76% at 20 °C and 30 °C, respectively (*p* < 0.05). However, a root-zone temperature of 15 °C did not cause significant changes in the leaf PSII quantum yield.

### 2.4. Response of Lignan Content Under Different RZT Conditions

**Figure 4:**
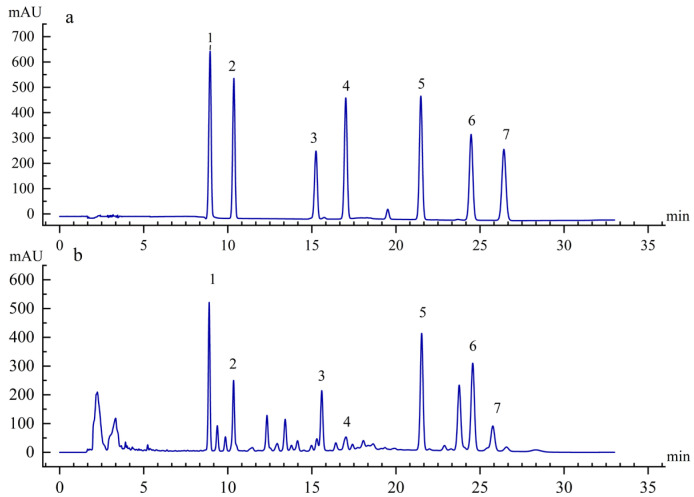
Mixed standard (**a**) and sampled (**b**) by high-performance liquid chromatography (HPLC). (1) schisandrol A, (2) schisandrol B, (3) schisantherin A, (4) schisanhenol, (5) schisandrin A, (6) schisandrin B, (7) schisandrin C.

**Figure 5:**
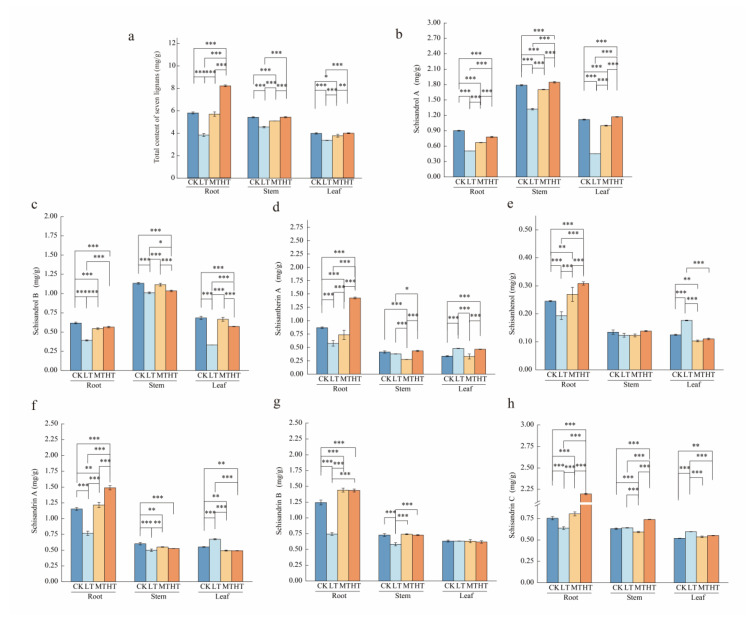
Root, stem, and leaf accumulation of seven lignans (**b**–**h**), and total accumulation (**a**) under different root-zone temperatures for *S. chinensis*. The data are expressed as mean ± SD (*n* = 3). Significant differences between root-zone temperature treatments are indicated by *** (*p* < 0.001) and ** (*p* < 0.01), * p < 0.05 above the line.

Seven major *S. chinensis* lignans were quantified by HPLC (Figure 4). Compared to the control (CK), the 15 °C RZT treatment significantly suppressed lignan accumulation in the roots, stems, and leaves, resulting in reduced lignan content across all organs. At a 20 °C RZT, lignan synthesis in the aboveground parts was inhibited, while the roots showed no significant changes. At a 30 °C RZT, the lignan content reached its peak in the roots. Although the lignan content increased in aboveground parts, this increase was not statistically significant compared to CK (Figure 5a). The content of specific lignans varied across plant components (Figure 5b–h). Specifically, compared to control roots, the levels of individual lignans decreased significantly at a 15 °C RZT. At a 20 °C RZT, schisandrol A, schisandrol B, and schisantherin A showed significantly lower levels. Conversely, the root levels of all individual lignans, except schisandrol A and schisandrol B, increased significantly with rising RZT. Compared to the stems in the control group, at a 15 °C RZT, the synthesis of individual lignans was inhibited. At a 30 °C RZT, the schisandrin C content peaked in roots, increasing by 16.94% compared to CK. In leaves, schisantherin A, schisanhenol, schisandrin A, and schisandrin C levels increased by 41.16%, 41.39%, 22.37%, and 14.65%, respectively, compared to the control. In contrast, when the RZT was 15 °C, schisandrol A and schisandrol B decreased by 59.44% and 51.55%.

### 2.5. PLS-DA Analysis of Secondary Metabolites

**Figure 6:**
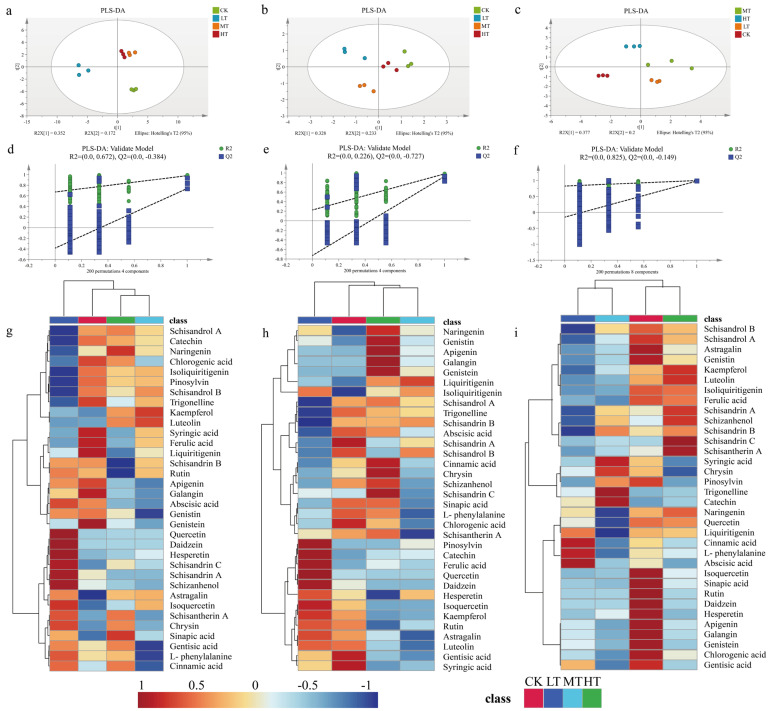
Changes in the content of secondary metabolites in the roots (**a**,**d**,**g**), stems (**b**,**e**,**h**), and leaves (**c**,**f**,**i**) of *S. chinensis* after 30 d (*n* = 3) of treatment at different temperatures in the root zone.

To investigate the impact of RZT variations on secondary metabolite accumulation in *S. chinensis*, we profiled metabolites using partial least squares discriminant analysis (PLS-DA) and hierarchical clustering heatmaps. The multivariate analysis revealed significant changes in metabolite profiles under different root-zone temperature regimes, with temperature-dependent accumulation patterns observed across experimental groups (Figure 6). Principal component analysis (PCA) of roots showed clear separation among treatments along the first two principal axes, accounting for 37.7% and 20.0% of total variance, respectively (Figure 6a). PLS-DA models for stems and leaves explained 52.4% and 56.1% of the variance, respectively, between the CK and temperature-treated groups (Figure 6b,c). Model validation through 200 permutation tests confirmed statistical robustness, with positive regression line slopes and Q2 intercepts < 0.05, eliminating concerns regarding overfitting or underfitting (Figure 6e–g). These validated models were then used to screen differential metabolites.

### 2.6. Screening of Phenolic Differentially Expressed Metabolites (DEMs)

**Figure 7:**
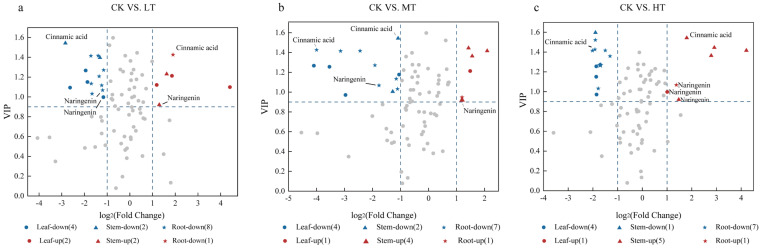
Volcanogram of differential metabolites of *S. chinensis* after 30 days of exposure to 15°C (**a**), 20 °C (**b**) and 30 °C (**c**) RZT (*n* = 3).

Metabolic network structural analysis of phenolic DEMs was performed using fold change (FC) and variable importance in projection (VIP) criteria. DEMs were defined as metabolites meeting |log2FC| ≥ 1 and VIP ≥ 0.9 thresholds, with differential expression patterns visualized through volcano plots (Figure 7). Our analysis revealed temperature-dependent modulation of secondary metabolite biosynthesis pathways. Likewise, we found 15 DEMs (two up- and one down-regulated for leaves, one up- and two down-regulated for stems, and seven down-regulated for roots) for the CK vs. MT treatments (Figure 7b). For the CK vs. HT group, we observed 19 DEMs (2 up-regulated and 4 down-regulated in leaves; 3 up-regulated and 3 down-regulated in stems; and 7 down-regulated in roots) (Figure 7c).

### 2.7. Structural Analysis of Secondary Metabolism Network

**Figure 8:**
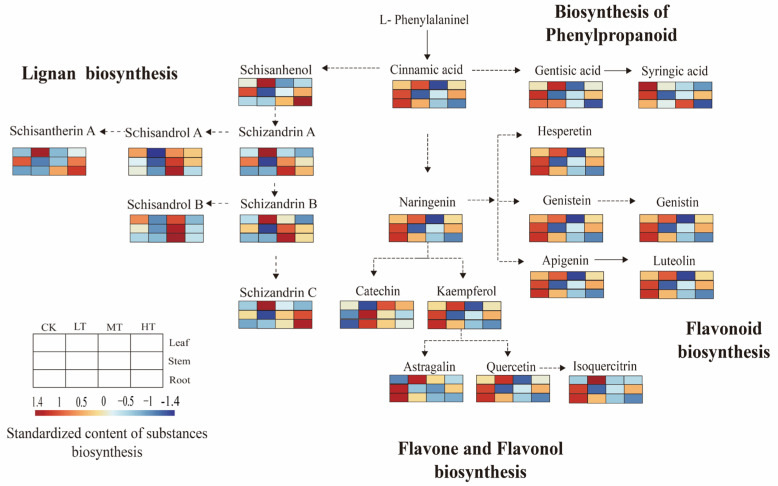
Metabolic pathway network of secondary metabolites in *S. chinensis* root zones under temperature stress. Color gradients reflect normalized metabolite concentrations (red: high abundance; blue: low abundance). Dashed and solid lines denote indirect and direct metabolic conversions, respectively, with arrows indicating biochemical transformation directions.

To investigate changes in metabolic processes in *S. chinensis* tissues under different RZTs, we mapped the identified differentially expressed metabolites (DEMs) and seven lignans using KEGG pathway analysis. The secondary metabolic network focused on four pathways: phenylpropanoid biosynthesis, flavonoid biosynthesis, flavonol biosynthesis, and lignan biosynthesis (Figure 8).

The results demonstrated significant temperature-dependent accumulation patterns. At a 15 °C RZT, cinnamic acid and most flavonoids showed elevated accumulation in leaves compared to CK, with synthesis inhibition observed at 20 °C and 30 °C RZTs. Schisanhenol accumulated preferentially in leaves at a 15 °C RZT, but showed suppressed synthesis in stems and roots. Root-specific lignan synthesis was enhanced at a 20 °C RZT. Schisandrol A, a key quality indicator, accumulated to high levels in the roots at a 30 °C RZT, whereas synthesis was inhibited when the RZT was 15 °C. These differential accumulation patterns may contribute to *S. chinensis*’ physiological adaptation to RZT variations.

## 3. Discussion

The root-zone temperature significantly influences plant development and physiological processes through its modulation of root growth and initiation [26,27]. Our results showed that *S. chinensis* plants exhibited significant growth inhibition when the RZT was 15 °C. Low-RZT conditions suppressed root developmental patterns and morphological architecture by altering the root growth rate, surface area expansion, cell elongation, and lateral root formation, ultimately reducing the overall growth rate and significantly decreasing organ biomass [28,29,30]. Notably, strawberry (*Fragaria × ananassa* (Weston) Duchesne ex Rozier) plants exposed to a 5 °C RZT displayed impaired growth and compromised fruit quality [31]. At a 20 °C RZT, *S. chinensis* showed enhanced shoot growth and increased leaf biomass, concomitant with root growth suppression. These findings align with previous reports demonstrating strong interactive effects between the RZT and cucumber (*Cucumis sativus* L.) growth parameters, including dry weight allocation, root elongation patterns, and leaf area expansion [32,33]. At an RZT of 30 °C, the root growth of *S. chinensis* was enhanced, while leaf biomass decreased. Xia et al. [34] demonstrated that an RZT of 30 °C significantly enhanced maize (*Zea mays* L.) growth relative to 24 °C (CK) and 36 °C (HT) conditions, with marked increases in plant height, stem diameter, root length, and dry matter accumulation. Compared to the C3 plant *S. chinensis*, maize, a C4 species, exhibits markedly divergent physiological responses to RZT. This discrepancy can be attributed to the inherent differences in their photosynthetic mechanisms, resource allocation strategies, and evolutionary adaptability [35]. The aboveground and underground parts of plants may exhibit differential responses to temperature that influence growth and resource allocation [36,37]. Changes in the root-zone temperature affect hormones like auxin and abscisic acid by impacting how the hormones are made and balanced in the root zone, which can lead to growth problems in different plant parts [38,39]. Research has demonstrated the impact of root zone temperatures on the transport of hormones from roots to aboveground parts [40]. The synthesis and upward transport of CTK and GA in the root are generally reduced at low root-zone temperatures, while the synthesis and transport of ABA are increased [41]. This differential response could account for the limited growth of certain vegetative organs of *S. chinensis* at both the 20 °C and 30 °C RZTs.

In addition to affecting plant growth, RZT also affects the production and enrichment of secondary metabolites in medicinal plants, thereby altering their quality characteristics [42,43]. The biosynthesis of rosmarinic acid and salvianolic acid B in *Salvia miltiorrhiza Bunge* is stimulated by specific environmental stresses, particularly low temperature [44]. Schisanhenol is one of the initial compounds synthesized in the lignan pathway, and schisandrol A serves as the standard index for the quality evaluation of *S. chinensis*. It is a representative dibenzocyclooctadiene lignan with significant medicinal value [8,45]. Schisanhenol synthesis was inhibited in the roots and the molecule accumulated in leaves at a 15 °C RZT. At 20 °C and 30 °C RZTs, accumulation occurred in the roots, while leaf synthesis was inhibited. Notably, the marker compound for quality (schisandrol A) accumulated in leaves and stems at a 30 °C RZT and its synthesis was inhibited at 15 °C RZT. High-temperature stress activates the biosynthesis of phenylpropanoids in plants, resulting in the accumulation of secondary metabolites. Compounds such as lignans play an important role in protecting cells from DNA damage [23,46]. Furthermore, elevated temperatures have been shown to facilitate the translocation of secondary metabolites from roots to shoots by means of activating the ABA (abscisic acid) signaling pathway, thereby enhancing antioxidant defense mechanisms [47]. However, the biological synthesis of dibenzocyclooctadiene lignans is still incompletely understood [12,48]. Whether there is reciprocal transformation between individual lignan compounds at different root-zone temperatures requires further research.

As a further product of the phenylpropanoid pathway, flavonoids have been shown to regulate auxin transport, promote pollen germination, and act as antioxidants [12,14]. In this study, the flavonoid content showed tissue specificity. Naringenin, which is associated with the biosynthesis of flavonoids and flavonols, showed higher enrichment in leaves at a 15 °C RZT, and its synthesis was inhibited at 20 °C and 30 °C RZTs. In previous studies, the content of naringenin in *Pinellia ternata* (Thunb.) Breit. was greatly increased under cold stress, and the accumulation of naringin was decreased under high-temperature stress [49]. Compared to the control, a 15 °C RZT upregulated the biosynthesis of cinnamic acid and lignans in leaves, resulting in their increased accumulation. Concurrently, flavonoid production via the naringenin node was significantly enhanced, leading to an increased flavonoid content. We hypothesize that the co-accumulation of lignans and flavonols substantially contributes to the defense and resistance of plants to stress under different RZTs [7,50,51,52].

The present study used a novel approach of conducting differential temperature control experiments on the aerial and subterranean parts of the plant. In this study, we investigated how the soil temperature affects the development of various vegetative organs of *S. chinensis*. The results revealed a suitable elevation in root-zone temperature was advantageous for enhancing the effective components of *S. chinensis*.

## 4. Materials and Methods

### 4.1. Plant Material and Root Zone Temperature (RZT) Treatments

*S. chinensis* seeds were obtained from Benxi, Liaoning Province, China. Following harvest, they were immersed in clean water, massaged to remove the pulp, and allowed to dry in the shade. The seeds were stored at 0–4 °C for 30 days after soaking for 24 h. They were then combined with wet dirt in a volume ratio of 1:3. Germination took place in climate-controlled chambers, 60% relative humidity, and temperatures of 25/15 °C. In the four-leaf stage, plants with consistent growth were divided into four groups and moved to the greenhouse. The aboveground temperature of seedlings was maintained at a constant 25 °C.

The administration of root-zone temperature treatments was conducted using a refrigerated circulating water bath system, maintaining constant temperatures of 15 °C (LT), 20 °C (MT), and 30 °C (HT). Control plants (CK) were maintained under ambient root zone temperatures equivalent to the shoot environment conditions. On day 30 after treatment initiation, plants were destructively sampled for comprehensive growth analysis. Root systems were carefully excised and subjected to two sequential washes in distilled water to remove adhering substrate particles. Cleaned roots were scanned using a high-resolution root imaging system, and architectural parameters were analyzed using LA-S root analysis software (Wan Sheng Hangzhou).

### 4.2. Determination of Chlorophyll Content and Photosynthetic Parameters

After 30 days of exposure, the third totally enlarged leaf was picked randomly from the top of each group, and the parameters related to photosynthesis in *S. chinensis* seedlings were determined using a portable photosynthetic assay system (Li-6400, LI-COR Inc., Lincoln, NE, USA). The estimation of the maximum quantum yield (Fv/Fm), the actual quantum yield [Y(II)], and the photosynthetic electron transport rate (ETR) of the leaves was accomplished by means of a portable modulated chlorophyll fluorescence meter (PAM-2500, Walz, Effeltrich, Germany).

In order to extract pigments, three plant leaves (100 mg, WM) were soaked in 5 mL dimethyl sulfoxide at 60 °C for 2 h. The degree of light absorption by the supernatant at 480, 649, and 665 nm was determined using a spectrophotometer (723PC, Shanghai Jinghua Technology Co., Ltd., Shanghai, China). Absorbance was calculated using Wang et al.’s [53] formula.

### 4.3. Analysis of Secondary Metabolites

#### 4.3.1. Determination of Lignan Content

The lignans of *S*. *chinensis* were extracted using the method of Mocan et al. [54], with a slight modification. The 100 mg (dry basis) sample was extracted with 5 mL of high-performance liquid chromatography-grade methanol for 45 minutes, and the supernatant was dried.

Each sample was then redissolved in 1 mL methanol and filtered through a 0.22 µm nylon membrane. The filtrated extracts were then analyzed by HPLC. The analysis was performed using a Thermo Vanquish Core liquid phase system with an Agilent TC-C18 column (4.6 × 250 mm, 5 µm, Agilent Inc., Santa Clara, CA, USA). According to Chang et al. [55], the HPLC analysis used deionized water (A) and acetonitrile (B) as mobile phases. The method for gradient elution was executed as follows: The initial 0–25 min of the process was conducted at a flow rate of 55–75% B. This was followed by a 25–30 min period during which the flow rate was adjusted to 75–75% B. The subsequent 30–35 min involved a transition to 75–55% B, with a constant flow rate of 1 mL/min. The system temperature was maintained at 30 °C, and the injection volume was set to 20 μL. The wavelength of the detectors was set at 254 nm, and the total duration of the procedure was 35 min. Standard solutions with varying concentrations were prepared for the quantification of lignans. We used standard calibration curves of seven lignan standards (≥99%, Chengdu Must Biotechnology Co., Ltd., Chengdu, China) to compute the results.

#### 4.3.2. LC-MS Analysis of Phenolic Compounds

Metabolic products were isolated from samples of roots, stems, and leaves using LC-MS as reported by Liu et al. [56]. Using an electronic balance, weigh the biological sample (DM), add 3 mL of 70% methanol, and sonicate (100 kHz) at 40 °C for 45 min; then, mix and evaporate using a rotary evaporation apparatus twice. For UPLC-MS/MS analysis, all samples were diluted in 1 mL with 70% methanol, sandwiched between a 0.22 µm screen, and placed in an injection bottle.

The separation system consisted of an HPLC equipped with an LC-20 AD pump, temperature control, a column temperature box, an SIL 20 A autosampler (Watts, Shanghai, China), and a BEHC 18 column (1.7 um, 2.1 mm × 50 mm) equipped with the same type of precolumn. The experiment kept the column at 30 °C. We used a mixture of 0.05% acetic acid and water (A) and 0.05% acetic acid and acetonitrile (B) as the solvent system. The flow rate was 0.25 mL/min. After 23 min, there was 5% B and 95% B, and after 25 min, there was 95% B and 5% B. The mass spectrometer operates in positive electron mode in the range 50 to 1000 *m*/*z*.

### 4.4. Statistical Analysis

The data were analyzed using SPSS Statistics (IBM SPSS Statistics, v. 25.0). To analyze the differences in plant growth and phenolic compounds at different root-zone temperatures, one-way analysis of variance (ANOVA) and least significant difference (LSD) tests were used. We used SIMCA 14.1 (Umetrics, Umeö, Sweden) for multivariate statistical analysis, including partial least squares discriminant analysis (PLS-DA), to compare secondary metabolites in the CK, LT, MT, and HT treatments. To screen for differentially expressed metabolites (DEMs), variable importance in prediction (VIP) > 0.9 and |log2 fold change (FC)| ≥ 1 were used. Pathways were constructed from the Kyoto Encyclopedia of Genes and Genomes (KEGG) pathway database.

## 5. Conclusions

This study investigated the response of different organs of *S. chinensis* to various root-zone temperatures (RZTs). The results indicated that low-temperature treatment (a 15 °C RZT) significantly inhibited plant growth. The 20 °C RZT regime promoted shoot growth in *S. chinensis*, whereas the 30 °C RZT regime stimulated root growth. The physiological pathway of photosynthesis was affected by different RZT treatments (15 °C, 20 °C, and 30 °C) due to their regulation of root growth and development, resulting in changes in chlorophyll fluorescence parameters. The regulation of different RZT levels on the lignan content in the roots, stems, and leaves of *S. chinensis* demonstrated notable organ specificity. A 15 °C RZT inhibited the synthesis of *S. chinensis* lignans. At a 20 °C RZT, lignan synthesis in the aboveground parts was inhibited, with no significant changes observed in the roots. Conversely, at 30 °C, the lignan content was highest in the roots. Although there was an increase in the aboveground parts, the difference compared to the control group was not significant. Notably, the 30 °C RZT treatment significantly promoted the synthesis of Schisandrol A, increasing its accumulation by 43.88% in aboveground parts. At the metabolic level, the dynamic changes in the content of certain phenolic compounds, such as cinnamic acid and naringenin, indicate that lignans and flavonols co-accumulate under varying root-zone temperatures to protect plants from stress. Studies have demonstrated that a moderate increase in RZT is beneficial for enhancing the medicinal value of the aboveground parts of *S. chinensis*.

## Figures and Tables

**Table 1:** Photosynthetic performance and photosynthetic pigmentation of *S. chinensis* under different root zone temperature treatments.

	Pn (μmol m^−2^ s^−1^)	Gs (μmol m^−2^ s^−1^)	Ci (mmol m^−2^ s^−1^)	Tr (mmol m^−2^ s^−1^)	Chla (mg/g)	Chlb (mg/g)	Car (mg/g)	Chla + chlb
CK	2.13 ± 0.21d	0.035 ± 0.0041b	302.4 ± 20.82a	0.87 ± 0.13c	0.76 ± 0.14c	0.47 ± 0.05b	0.24 ± 0.02c	1.23 ± 0.09c
LT	3.72 ± 0.32c	0.037 ± 0.0042b	213.8 ± 36.49b	1.16 ± 0.13b	1.45 ± 0.01ab	0.52 ± 0.05b	0.39 ± 0.01b	1.97 ± 0.05b
MT	5.87 ± 0.28b	0.050 ± 0.0039a	291.6 ± 49.93a	1.72 ± 0.13a	1.41 ± 0.10b	0.53 ± 0.05b	0.39 ± 0.03b	1.94 ± 0.15b
HT	6.71 ± 0.27a	0.037 ± 0.0038b	218.4 ± 27.54b	1.18 ± 0.09b	1.60 ± 0.06a	0.62 ± 0.03a	0.45 ± 0.02a	2.22 ± 0.09a

Values are presented as the mean ± standard deviation (*n* = 6). Means with the same letter (a, b, c, d) are not significantly different (*p* < 0.05).

## Data Availability

The original contributions presented in this study are included in the article. Further inquiries can be directed to the corresponding author(s).
